# Population based prostate cancer screening in north Mexico reveals a high prevalence of aggressive tumors in detected cases

**DOI:** 10.1186/1471-2407-9-91

**Published:** 2009-03-24

**Authors:** Lauro S Gomez-Guerra, Margarita L Martinez-Fierro, Valeria Alcantara-Aragon, Rocio Ortiz-Lopez, Rebeca T Martinez-Villarreal, Idelma B Morales-Rodriguez, Raquel Garza-Guajardo, Marco A Ponce-Camacho, Augusto Rojas-Martinez

**Affiliations:** 1 School of Medicine and University Hospital, Universidad Autonoma de Nuevo Leon, Monterrey, Mexico

## Abstract

**Background:**

Prostate Cancer (PCa) is the second most frequent neoplasia in men worldwide. Previous reports suggest that the prevalence of PCa in Hispanic males is lower than in Africans (including communities with African ancestry) and Caucasians, but higher than in Asians. Despite these antecedents, there are few reports of open population screenings for PCa in Latin American communities. This article describes the results of three consecutive screenings in the urban population of Monterrey, Mexico.

**Methods:**

After receiving approval from our University Hospital's Internal Review Board (IRB), the screening was announced by radio, television, and press, and it was addressed to male subjects over 40 years old in general. Subjects who consented to participate were evaluated at the primary care clinics of the University Health Program at UANL, in the Metropolitan area of Monterrey. Blood samples were taken from each subject for prostate specific antigen (PSA) determination; they underwent a digital rectal examination (DRE), and were subsequently interviewed to obtain demographic and urologic data. Based on the PSA (>4.0 ng/ml) and DRE results, subjects were appointed for transrectal biopsy (TRB).

**Results:**

A total of 973 subjects were screened. Prostate biopsy was recommended to 125 men based on PSA values and DRE results, but it was performed in only 55 of them. 15 of these biopsied men were diagnosed with PCa, mostly with Gleason scores ≥ 7.

**Conclusion:**

Our results reflect a low prevalence of PCa in general, but a high occurrence of high grade lesions (Gleason ≥ 7) among patients that resulted positive for PCa. This observation remarks the importance of the PCa screening programs in our Mexican community and the need for strict follow-up campaigns.

## Background

According to the World Health Organization, cancer causes 7 million deaths worldwide every year and prostate cancer (PCa) is the second most frequent malignant neoplasia. PCa represents the second cause of death due to cancer among Mexican men [1]. As life expectancy of the general population increases in Latin American countries, a positive correlation with the incidence of PCa is also expected [2]. Screenings in these populations are scarce, although studies in Hispanic populations living in the United States report that this heterogeneous ethnic group has medium risk of PCa when compared with other groups. Early diagnosis of PCa can increase the likelihood of cure, although screening asymptomatic men for PCa is controversial due to problems with the screening test (e.g., sensitivity and specificity) as well as the lack of evidence that screening affects population mortality from disease [3-5]. To better understand the prevalence of risk factors and biopsy prevalence of disease, we conducted a population-based PCa screening using PSA and DRE in the metropolitan area of Monterrey, Mexico. We describe the results of three screening efforts.

## Methods

This study was approved by the Ethics Committee of the University Hospital, from the Universidad Autonoma de Nuevo Leon and was thereafter announced by local radio, television and printed press. Men over age 40 who lived in the metropolitan area of Monterrey, Mexico were eligible to participate. Subjects were screened in the primary health centers of the University Health Program UANL located in Monterrey, Apodaca, and Guadalupe (Monterrey's Metropolitan area). Subjects were informed of the potential but unproven benefits and harms associated to their participation in the study. No exclusion criteria were considered for this study. From each participating subject who granted his informed consent, a venous blood sample was obtained for PSA determination, a questionnaire consisting of demographic and urologic data (American Urological Association Symptom Index: AUA-SI) was administered, and a DRE was performed by an urologist or urology resident. If the serum PSA level was equal to or above 4.0 ng/ml or the DRE was abnormal, a transrectal biopsy (TRB) was recommended. Before biopsy, patients were premedicated with 1 gr/day of ciprofloxacin for three days and an enema (450 mg sodium citrate/45 mg lauryl sulfoacetate). The transrectal ultrasound guided biopsies (TRUS) of the prostate were performed under local anesthesia with xilocaine gel using the biopsy system ASAP™ with Channel Cut™ 18 GA needles (length 21 cm with a cutting channel of 17 mm); at least six cylinders were obtained from each patient, this number corresponds to the sextant technique, a standard method practiced in Mexico. The prostatic tissue obtained was fixed in 3.7% neutral-buffered formalin for at least 3 hours, processed overnight and embedded in paraffin according to the standard protocol. Two 5-μm thick sections were performed, and stained with the Hematoxilin and Eosin stain. The slides were evaluated for prostatic carcinoma by two pathologists. All the neoplasms were graduated using the Gleason score. Screenings were performed on July 2004, July 2005, and February 2006.

## Results

A total of 973 subjects were screened in the study; 709 were entered the study in 2004, 225 in 2005, and 39 in 2006. Most of the screened subjects were from the state of Nuevo Leon: 40% from Guadalupe followed by Monterrey, Apodaca and San Nicolas districts (31, 12 and 9% respectively). Two percent of the subjects were from other districts of Nuevo Leon and 1% from the neighbor states of Coahuila, Tamaulipas, and Veracruz. The mean age of participants at the time of the first screening was 61.9 (range: 40–98). Anthropometric measures indicated 76.5% of the men were overweight with a Body Mass Index (BMI) above or equal to 25, 21% were had a normal BMI (20–24.9) and 2.5% were underweight (BMI below 20). A total of 54% of subjects presented with mild (0–7 AUA-SI) obstructive urinary symptoms while 46% had moderate (8–19 AUA-SI) or severe (20–35 AUA-SI) obstructive symptoms. Table 1 shows age, BMI, and urologic data for the studied population, compared with the data obtained from subjects diagnosed with PCa.

**Table 1:** Age, Body Mass Index, and Urologic Data for the General Population Screened compared with data from PCa Subjects.

Attribute	General Population Screened	Subjects with PCa
	
	*n = 958*	*n = 15*
Age Mean (range)	61.9 (40–98)	69.6 (55–84)
Mean BMI	27.98	26.57
Mean AUA score	8.72	10.4
Mild Urinary Symptoms (%) *	53.94	33.33
Moderate Urinary Symptoms (%)^#^	33.82	53.33
Severe Urinary Symptoms (%)^~^	12.24	13.33

* AUA Symptom Index Score Range from 0 to 7.

^# ^AUA Symptom Index Score Range from 8 to19.

^~ ^AUA Symptom Index Score Range from 20 to 35.

In the 2004 screening, we found 77 subjects with PSA levels above 4 ng/ml; and one subject with PSA level <4, but an abnormal DRE. Of these subjects, 41 elected to undergo TRB. Pathological evaluation found that 13 (31.7%) of these men had cancer, 92.3% being high grade (Gleason ≥ 7) disease. (Table 2)

**Table 2:** PSA levels for subjects in the screenings of 2004, 2005 and 2006, cases of PCa found, and their Gleason scores.

PSA levels (ng/ml)	2004 screening			2005 screening			2006 screening		
	
	Number of subjects in this range	PCa cases in this range*	Gleason Scores	Number of subjects in this range	PCa cases in this range*	Gleason Scores	Number of subjects in this range	PCa cases in this range*	Gleason Scores
0.1 – 4.0	620	1	7	175	0	-	29	0	-
4.1 – 8.0	53	2	7,8	22	0	-	5	0	-
8.1–12.0	10	4	7,8,6,9	6	0	-	4	1	8
> 12.0	14	6	7,9,7,9,9,8	9	1	9	1	0	-

Total	697 •	13	-	212 †	1	-	39	1	**-**

• PSA could not be measured for 12 subjects due to insufficient blood sample.

† PSA could not be measured for 13 subjects due to insufficient blood sample.

* Prostate cancer cases found only within the 55 biopsies performed in these screenings.

During the 2005 screening effort, we detected 37 men with PSA levels above 4 ng/ml and 11 biopsies were performed; one of them (9.9%) ultimately diagnosed Gleason 9, metastasic PCa. (Table 2)

In 2006 we found ten subjects with PSA levels above 4 ng/ml. Three biopsies were performed and we found one subject (33.33%) with PCa, Gleason score 8. The PSA determination levels for these subjects are presented on table 2.

**Figure 1:**
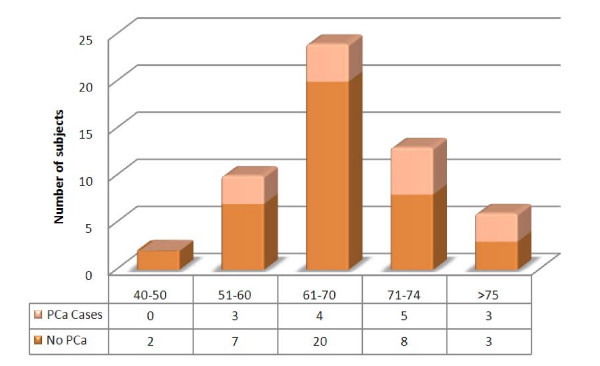
**Age distribution of biopsied subjects (n = 55)**.

Overall, fifty-five core biopsies from 124 subjects with suspicion of PCa (by PSA and DRE criteria) were submitted for pathological evaluation and a total of 15 cases of PCa were found in the screenings. The typical features of prostatic carcinoma like a single epithelial layered gland, huge nucleolus and conspicuous nucleoli were observed; some of the glands showed back to back pattern of growth, lymphatic and vascular infiltration; 14 of the carcinomas (93%) had Gleason scores equal or above 7. Sixty seven percent of the subjects with PCa had moderate to severe obstructive symptoms according to their AUA-SI scores. None of the men with prostate cancer had a history of PCa in their families, and only 2 had a family history of other types of cancer (Colon Ca, and Breast Ca). Most of the subjects with PCa (66.6%) were overweight, with a Body Mass Index (BMI) equal or above 25. Only one of these subjects was reported as smoker at the time of the interview and seven (46.6%) were taking nutritional supplements, mostly multivitamin compounds. Table 1 shows Age, BMI and AUA data for these subjects compared with general population. 98 screened patients were older than 75 years (10.07%). The age distribution of subjects that underwent biopsy (Figure 1) shows that PCa was diagnosed in 12/37 subjects from 40 to 74 years old (32.43%), while this tumor was diagnosed in 3/6 subjects older than 75 years old (50.0%).

**Figure 2:**
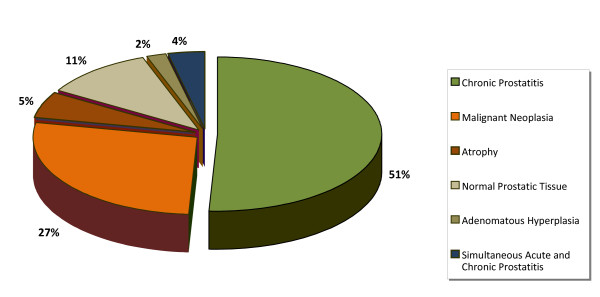
**Histologic findings in TRB tissue samples from all the screenings (n = 55)**.

The only histological type of PCa detected was adenocarcinoma (Figure 2). As for the other histological findings, the most common was chronic prostatitis (51%), followed by malignant neoplasia (27%), benign prostatic tissue (11%), atrophy (5%), chronic prostatitis with secondary acute prostatitis (4%), and prostatic adenomatous hyperplasia (2%). Chronic prostatitis, being the most common diagnosis, was found in association to benign prostatic tissue in 86% of the samples, followed by 7% in hyperplasic prostatic tissue, 5% atrophic tissue, and finally accompanied by prostatic infarct in 2%.

Regarding the prostate biopsies performed for the study, 81.8% were due to elevated PSA, while 18.2% were exclusively due to an abnormal DRE. Table 3 displays the false positive and negative rates as well as the predictive values, sensitivity and specificity rates of these tests according to our results.

**Table 3:** Effectiveness of PCa diagnosis: Accuracy of PSA and DRE.

MEASURE	PSA	DRE
False Positive Rate	0.77	0.18
False Negative Rate	0.07	0.8
Sensitivity	93.30%	20%
Specificity	22.50%	82%
Positive Predictive Value	31%	10%
Negative Predictive Value	90%	91%

## Discussion

Since PCa is one of the main causes of death among men worldwide, availability of screening tests make early detection an attractive public health option for developed countries. The American Cancer Society recommends offering the combination of PSA and DRE annually to men over age 50 and to men over age 45 for subjects with additional risk factors such as a family history of PCa or African American ethnicity [6]. Our series is the first report of a public screening for PCa in Mexico, most of the participants did not have antecedents of PSA screening neither prostate biopsy for cancer detection. This article illustrates both the ability of such an effort to detect prostate cancer cases of whom many had high tumor grades, as well as the surprisingly high proportion (46%) of moderate to severe obstructive symptoms among Mexican men. The U.S. Preventive Services Task Force (USPSTF) Recommendation Statement established a threshold of age at 75 for PCa screening; USPSTF recommends against routinely providing the screening to asymptomatic patients because found at least fair evidence that screening is ineffective or that harms outweigh benefits [7]. In Mexico, there is no experience about the impact of harms/benefit for early detection screening of PCa in men older than 75 years. In our study, a total of 98 patients were older than 75 years and PCa was diagnosed in 3/6 subjects that underwent biopsy (50%). This reflects the higher incidence of PCa in older men, and accordingly, we do not oversee the benefit of the screening for PCa in this group of men.

Only 15 cases of PCa were detected in the screenings. We are aware that this number may be affected by the compliance for biopsy in suspected subject, and additional factors, as the threshold criteria for serum PSA. Higher Gleason scores were common among men diagnosed with PCa and were not unsuspected, given the higher PSA levels among these men. Nonetheless, we also detected Gleason 7 prostate cancer in one of the subjects with a low PSA level. The occurrence of a high grade PCa with lower PSA levels, confirms the need for a serum marker with a higher sensitivity to diagnose this disease. DRE proved to be a screening test with low sensitivity (20%) and specificity (82%). PSA has a respectable sensitivity (93.3%), but a very low specificity (22.5%). These accuracy results support the plausibility of using both methods as complementary in screening efforts.

We found no important associations between known risk factors and diagnosis of PCa. The only risk factor present in the majority of PCa subjects in this study was age above 50 (94.73%) and the prevalence of PCa cases by age among biopsied men older than 70 years was higher than in the others age groups (53.33%).

Our results are dissimilar to many other screening series in which a substantial number of cases have Gleason 6 or lower grade disease [8,9]. In this series, the vast majority were Gleason 7 or higher. The explanation for this phenomenon is unknown. Possible reasons may include the low frequency of screenings in the population, but may also reflect the previously-noted lower prevalence yet higher grade of disease among Hispanic men. Regardless, it illustrates the importance and yet the challenge of a screening program in Mexico: the combination of low prevalence yet high aggressiveness would lend a greater interest in biomarkers related to disease prognosis, as even reasonably-good specificity rates may lead to unacceptable rates of unnecessary screening and biopsy.

Some limitations for this study must be recognized. A threshold of serum PSA of 4.0 ng/ml was established as criterion for biopsy. Is now clear that the 2.5 ng/ml value of serum PSA is a better threshold for PCa detection, but this study was designed and conducted in a period in which this PSA serum concentration was under study as a parameter for the screenings [7,10]. The present study does not consider the possible effect of concurrent prostate infections and benign prostatic hyperplasia on the serum PSA concentrations in the screened subjects, as has been described [11-13]. An underestimation of cancer diagnosis in the biopsies may be due to the use of the sextant biopsy technique, the standardize method used in screening cancer programs in Mexico. It has been showed that increased numbers of biopsy cylinders per biopsied patient improve the chance for detection of prostate malignancies in suspected individuals [14-16]. Empiric biopsies in subjects without clinical indication for prostate biopsy render a better estimation of the actual PCa prevalence in any population. We did not performed empiric biopsies in our study. Finally, we recognize a low compliance for the biopsy procedure (44.0%) in our report, but it maybe reflects the historical compliance observed in the Service of Urology of our University Hospital, in which the compliance is 56.6%.

## Conclusion

PSA and DRE screening tests are associated with elevated false positive rates however the diagnostic yield of PCa is increased using both methods as complementary in screening efforts.

Our results reflect a low prevalence of PCa in general, but a high occurrence of high grade lesions (Gleason ≥ 7) among patients that resulted positive for PCa. This observation remarks the importance of the PCa screening programs in our Mexican community and the need for strict follow-up campaigns.

## Abbreviations

PCa: Prostate cancer; PSA: Prostate specific antigen; DRE: Digital rectal examination; TRB: Transrectal biopsy; TRUS: Transrectal ultrasound; BMI: Body Mass Index; USPSTF: U.S. Preventive Services Task Force.

## Competing interests

The authors declare that they have no competing interests.

## Authors' contributions

The contribution of each author was substantial for the study, LSG participated in the implementation of the screening campaign and he performed the digital rectal examination and was in charge of the biopsies. MLM was involved in the samples and data collection, processing of the samples, screening coordination, and in the preparation of the manuscript. VAA was involved in the organization, data processing and preparation of the article. ROL participated in the planning of the screening campaign and sample collection. RTM was enrolled in the campaign organization, database registry, and patient contacting. IBM collected samples and interviewed subjects for the epidemiologic and urologic database. RGG and MAP were responsible for the pathologic evaluation of the biopsies. ARM was in charge of the general planning and coordination of the screening campaign and the completion of this study. He helped to write the article and did a careful revision of the manuscript.

All authors read and approved the final manuscript.

## Pre-publication history

The pre-publication history for this paper can be accessed here:

http://www.biomedcentral.com/1471-2407/9/91/prepub

## References

[B1] Sistema Nacional de Información en Salud: Base de datos 2005 http://sinais.salud.gob.mx/mortalidad/

[B2] Programa de Acción: Cáncer de Próstata. Centro Nacional de Vigilancia Epidemiológica. Secretaría de Salud de México 2001 http://bibliotecas.salud.gob.mx/gsdl/collect/publin1/index/assoc/HASH18a3.dir/doc.pdf

[B3] Harris R Lohr KN Screening for prostate cancer: an update of the evidence for the U.S. Preventive Services Task Force Ann Intern Med 2002 137 11 917 929 12458993 10.7326/0003-4819-137-11-200212030-00014

[B4] Krahn MD Mahoney JE Eckman MH Trachtenberg J Pauker SG Detsky AS Screening for prostate cancer. A decision analytic view JAMA 1994 272 10 773 780 10.1001/jama.272.10.773 7521400

[B5] Kramer BS Brown ML Prorok PC Potosky AL Gohagan JK Prostate cancer screening: what we know and what we need to know Ann Intern Med 1993 119 9 914 923 7692780 10.7326/0003-4819-119-9-199311010-00009

[B6] Society AC Detailed guide: Prostate cancer http://www.cancer.org/docroot/CRI/content/CRI_2_4_3X_Can_prostate_cancer_be_found_early_36.asp

[B7] Screening for prostate cancer: U.S. Preventive Services Task Force recommendation statement Ann Intern Med 2008 149 3 185 191 2008/08/06 edition 18678845 10.7326/0003-4819-149-3-200808050-00008

[B8] Hoedemaeker RF Kwast TH van der Boer R de Koning HJ Roobol M Vis AN Schroder FH Pathologic features of prostate cancer found at population-based screening with a four-year interval J Natl Cancer Inst 2001 93 15 1153 1158 10.1093/jnci/93.15.1153 11481387

[B9] Roobol MJ Kranse R de Koning HJ Schroder FH Prostate-specific antigen velocity at low prostate-specific antigen levels as screening tool for prostate cancer: results of second screening round of ERSPC (ROTTERDAM) Urology 2004 63 2 309 313 discussion 313–305. 10.1016/j.urology.2003.09.083 14972478

[B10] Thompson IM Pauler DK Goodman PJ Tangen CM Lucia MS Parnes HL Minasian LM Ford LG Lippman SM Crawford ED Prevalence of prostate cancer among men with a prostate-specific antigen level < or = 4.0 ng per milliliter N Engl J Med 2004 350 22 2239 2246 10.1056/NEJMoa031918 15163773

[B11] Hedelin H Johansson N Stroberg P Relationship between benign prostatic hyperplasia and lower urinary tract symptoms and correlation between prostate volume and serum prostate-specific antigen in clinical routine Scand J Urol Nephrol 2005 39 2 154 159 10.1080/00365590510007685 16019770

[B12] Simardi LH Tobias-MacHado M Kappaz GT Taschner Goldenstein P Potts JM Wroclawski ER Influence of asymptomatic histologic prostatitis on serum prostate-specific antigen: a prospective study Urology 2004 64 6 1098 1101 10.1016/j.urology.2004.08.060 15596176

[B13] Pannek J Marks LS Pearson JD Rittenhouse HG Chan DW Shery ED Gormley GJ Subong EN Kelley CA Stoner E Influence of finasteride on free and total serum prostate specific antigen levels in men with benign prostatic hyperplasia J Urol 1998 159 2 449 453 10.1016/S0022-5347(01)63946-6 9649261

[B14] Eskew LA Bare RL McCullough DL Systematic 5 region prostate biopsy is superior to sextant method for diagnosing carcinoma of the prostate J Urol 1997 157 1 199 202 discussion 202-193. 10.1016/S0022-5347(01)65322-9 8976250

[B15] Brossner C Madersbacher S Bayer G Pycha A Klingler HC Maier U Comparative study of two different TRUS-guided sextant biopsy techniques in detecting prostate cancer in one biopsy session Eur Urol 2000 37 1 65 71 10.1159/000020102 10671788

[B16] Stamatiou K Alevizos A Karanasiou V Mariolis A Mihas C Papathanasiou M Bovis K Sofras F Impact of additional sampling in the TRUS-guided biopsy for the diagnosis of prostate cancer Urol Int 2007 78 4 313 317 10.1159/000100834 17495488

